# Reply to “Comment on “Heat transfer fluids: amino acid anion ionic liquid based IoNanofluids with remarkable thermal conductivity and low viscosity” by A. Chandra, Y. S. Sistla, M. A. Ahmed, D. V. S. Vasireddy, N. Jaglan, N. K. Das, T. Banerjee and V. S. Sistla, *RSC Adv*. 2025, **15**, 23146” by Marzena Dzidaa, Anna Kolanowska, Krzysztof Cwynar, Katarzyna Kaczmarek

**DOI:** 10.1039/d6ra02431a

**Published:** 2026-07-08

**Authors:** Yamini Sudha Sistla

**Affiliations:** a Department of Chemical Engineering, Shiv Nadar Institution of Eminence Delhi NCR India yamini.sistla@snu.edu.in

## Abstract

Our article “Heat transfer fluids: amino acid anion ionic liquid based IoNanofluids with remarkable thermal conductivity and low viscosity” was commented on by Marzena Dzidaa *et al.* In the comment article Marzena Dzidaa *et al.* have raised some concerns, especially about the characterization of amino acid anion ionic liquids, MWCNTs, and IoNanofluids. This response article addresses and provides clarifications for the most critical comments.

## Abstract 2

Clarifications and additional analysis confirm the reliability of reported properties of AAIL and INF. Only revision is the identification of non-Newtonian behavior in INFs at shear rates below 100 s^−1^; above this limit, Newtonian behavior prevails.

## Graphical abstract

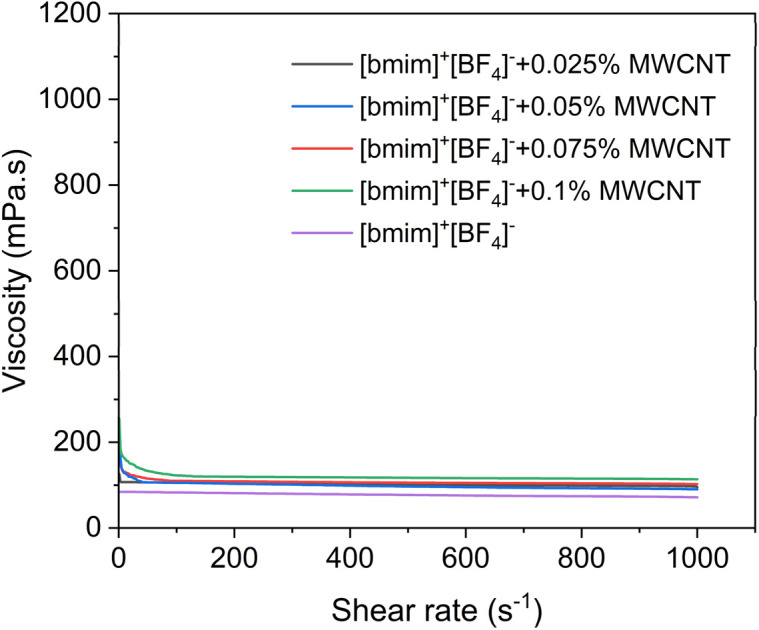

## Responses

Marzena *et al.* commented that “mostly, ILs are treated both as a continuous phase and as a surfactant in INFs. However, Chandra *et al.*^1^ studied the effect of 0.05 wt% loading of different surfactants on the density, uniformity of dispersion, *i.e.* size of agglomerates, and sedimentation stability of INFs composed of 0.05 wt% MWCNTs and 1-butyl-3-methylimidazolium tetrafluoroborate ([BMIm][BF4])”.

However, as mentioned in our original article, the microscopic analysis of INFs without the addition of surfactant showed agglomeration and non-uniform dispersion of the MWCNT. For instance, the large agglomerations of MWCNT in the [bmim][BF_4_] in the absence of surfactant can be clearly seen in Fig. 5(a) of our article (Chandra *et al.*),.^1^ Fig. 5 of our article indeed confirms the effect of various surfactants on the dispersion and reduction of agglomeration size of the MWCNT particles in the IL solvent.^1^ As discussed in the manuscript, Tween 80 (Fig. 5(d)) and CTAB (Fig. 5(e)) have enhanced the dispersion and fineness of nanoparticles. Furthermore, Fig. (10) of our article (Chandra *et al.*) also confirms the effectiveness of the surfactant on reducing the agglomeration size and improving dispersion of MWCNT particles in the INFs. Fig. (14) and (15) of our article also confirm the effect of surfactant on improving the colloidal stability of INF.^1^

Marzena *et al.* commented that, “However the works concerning the same type of INFs (studied INFs composed of (0.025–0.1 wt%) MWCNTs (diameter 37–53 nm, length was not specified, purity 95 wt%) and [BMIm][BF4]) were not cited”.

We would like to mention that we have certainly cited literature on MWCNT incorporated [BMIM][BF_4_] based INFs and some other ILs based INFs which we could get the full text articles. Also, we did not claim in our article that no work was done on MWCNT incorporated [BMIm][BF_4_] INFs.

Marzena *et al.* commented that “The aspect ratio has a substantial influence on the properties of CNTs, including thermal conductivity, which is the primary focus of the Chandra's *et al.* work. The greater aspect ratio leads to higher thermal conductivity of the INFs at a constant MWCNTs content.”

**Fig. 1:**
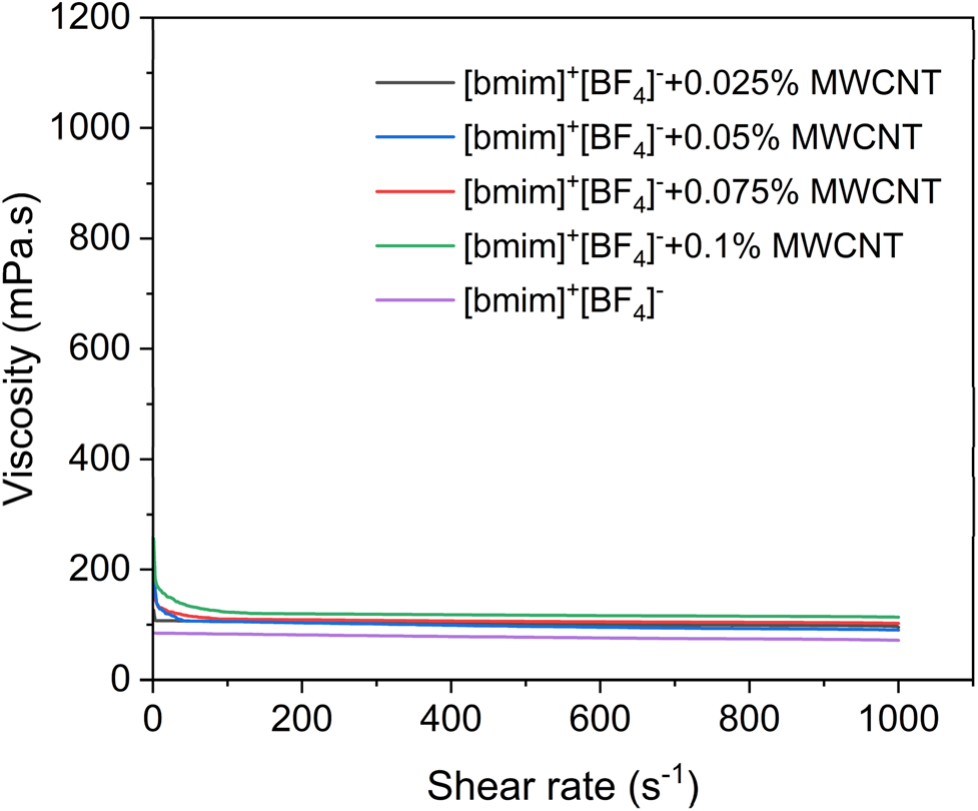
Fig. 6(a) of our article by re-scaling the *Y*-axis (to match with Creches *et al.*).

Certainly, the aspect ratio has influence on the properties of MWCNTs and the resulting INFs. Our original article clearly mentioned that we have used only one variety of MWCNT. The SEM micrograph (Fig. 1(a) and (b) of our article) clearly shows the diameter of the MWCNT ranging from 38 nm to 62.5 nm.^1^ As this was purchased from Sigma-Aldrich, it is obvious that the product code is 901019. The diameter observed by us from SEM matches with the diameter specifications of product code 901019 (diameter of 50–90 nm with average diameter of 65 nm). https://www.sigmaaldrich.com/IN/en/product/aldrich/901019. Length is not mentioned in the specification. Only the aspect ratio (>100) is mentioned. The aspect ratio (length/diameter) can provide the length of MWCNT from the diameter information. Since this was a purchased one but not a synthesized one by us in our lab, we did not feel the necessity to perform all the characterizations listed by Marzena *et al.* to confirm the purchased MWCNT is actually an MWCNT. The FTIR was done to confirm the presence/absence of any functional groups in MWCNT. FTIR of MWCNT was indeed performed using KBr pellet method. As this is standard, we did not feel like mentioning it explicitly.

Marzena *et al.* have compared the FT-IR spectra of MWCNTs reported by us (Chandra *et al.*) with those of reported by González-Domínguez *et al.* in Fig. 1 and they highlighted that both the FTIRs does not match.^1,2^

However, we would like to mention that we got the FTIR done for the purchased MWCNT. Marzena *et al.* have compared our FTIR with that of González-Domínguez *et al.* However, we would like to mention that González-Domínguez *et al.*, have synthesized the MWCNT and reported the FTIR of the MWCNT synthesized by them.^2^ We sincerely feel that this will not make a fair comparison. The Fig. 1(a) of Vinodh *et al.*, and 2 of Sharmeen *et al.*, also provide FTIR of MWCNT resembling with the FTIR reported by us in our article.^1,3,4^

**Fig. 2:**
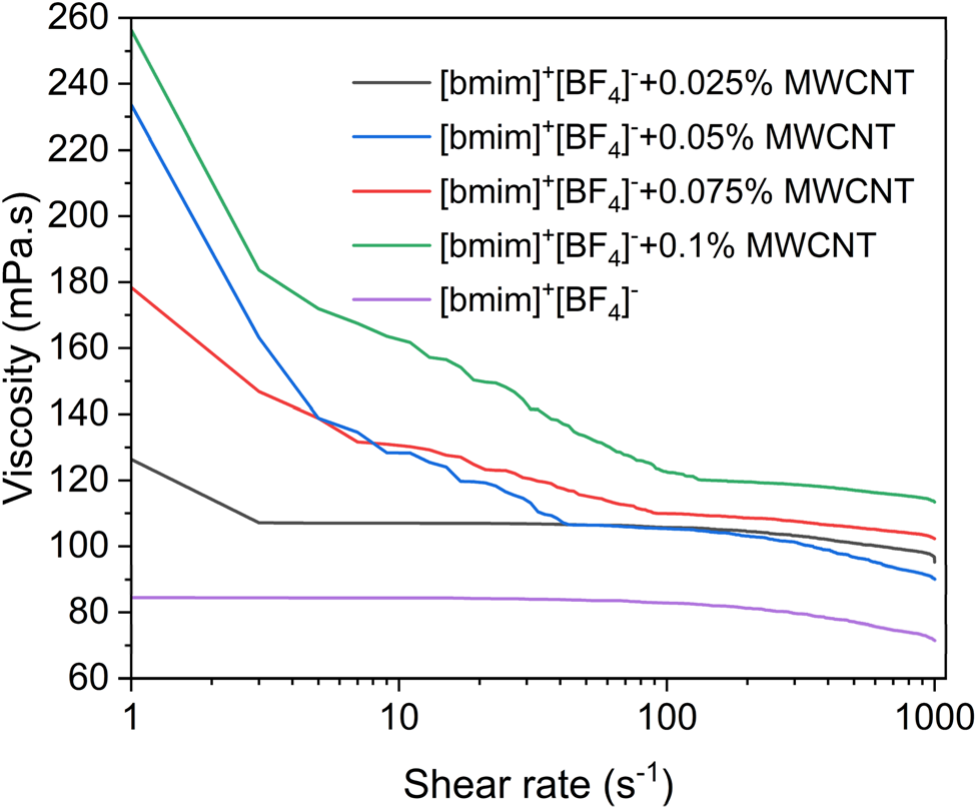
Fig. 6(a) of our article by re-scaling the *X*-axis to logarithmic (to match the *X*-axis of Fig. (6) of Cherecheş *et al.*).

Marzena *et al.* have compared the FTIR of [EMIm][Gly] reported by us (Chandra *et al.*) (black curve) and Bai *et al.* (red curve) in Fig. 2.^1,5^ Further, they compared the FTIR spectra glycine reported by us with that from spectral database for organic compounds.

We would like to mention that we have not synthesized glycine. As mentioned in the materials and methods section of our original article, we have purchased Glycine from Sigma Aldrich and got the FTIR done. Further, Chen *et al.* and Sabry *et al.* also reported FTIR of glycine which resembles the FTIR reported in our article.^1,6,7^ Further, Marzena *et al.* have compared the FTIR of [EMIm][Gly] reported by us with the FTIR of same IL reported by Bai *et al.*

We would like to mention that it is not compulsory that the FTIR of a compound synthesized by one research group to be “exactly same” as the FTIR of the same compound synthesized by other research group until the characteristic peaks confirming the molecular structure (fingerprint region) are matching. A slight shift in the peak position, intensity and sometimes broadening of the peaks are typically common. For example, the Han *et al.*, reported FTIR of four AAILs such as [BMIm][Ala], [BMIm][Lys], [BMIm][Arg], [BMIm][Gly].^8^ It can be clearly observed that the FTIR of these AAILs are different from our article and also different from Bai *et al.* as well which Marzena *et al.* have compared our FTIR with.^1,5,7^ The peaks, especially the N–H stretch region in the re-plotted FTIR by Marzena *et al.* appear broad. However, the Fig. 3(b) of our paper clearly shows sharp N–H stretch peak and absence of any OH peak corresponding to water. The FTIR of all the AAILs as reported in our article displayed all the characteristic peaks.

Marzena *et al.*, in Fig. 3. Of their comment article have compared the TGA curves of [EMIm][Gly] obtained by us (Chandra *et al.*) (black curve) and by Muhammad *et al.* (red curve).^1,9^ As discussed in our original article (Chandra *et al.*), the TGA pattern of ionic liquids depends on the type of anion, cation, cation–cation, anion–anion and cation–anion interactions. This phenomenon was also reported by other researchers as well. In addition, we believe that the TGA of AAIL reported by us in our article is accurate. Because, the TGA pattern was consistent with the concept of the presence of significant interionic hydrogen bonds in the AAIL (between “imidazolium cation–amino acid anion” and “amino acid anion–amino acid anion”) which was also reported by other researchers. For instance, the hydrogen bonds between carboxylic acid group of anion and ring hydrogen atoms of cation. Herrera *et al.* and Dong *et al.* have reported the presence of hydrogen bonds in AAILs through MD simulations and experiments.^10,11^

Therefore, the decomposition of AAIL would be gradual as reported in our article (Chandra *et al.*) instead of exhibiting stability till a high temperature and a sudden drop as observed for regular ILs (non-amino acid anion type) such as [bmim][BF_4_] *etc.* The TGA pattern of the AAILs as reported in our article is quite similar to the TGA of anthranilic acid based Imidazolium Ionic Liquids as reported by Gill *et al.*^1,12^ Also, Shahrom *et al.* also reported similar TGA pattern for an AAIL [VBTMA][Gly].^13^ Also, in the comparison plot, the way Marzena *et al.* plotted our data (black curve) is not fully consistent with the TGA plot in our manuscript. Furthermore, the pattern of TGA of [EMIm][Gly] reported by Muhammad *et al.* (red curve) which Marzena *et al.* have compared our data with does not show the presence of hydrogen bonds in the AAIL.^1,9^

Marzena *et al.* has highlighted that “The stability of AAILs up to 573 K was also not confirmed by differential scanning calorimetry (DSC). Chandra *et al.* found the first broad endotherm effect between 278–443 K having a peak at around 369–381 K for all four AAILs (see Fig. 4b in Chandra *et al.*).^1^ This effect was interpreted as the gradual phase transition due to melting. However, the authors reported the melting temperature of all AAILs as ∼372.15 K.” However, we did not understand this comment.

We have done TGA in a temperature range of 30–600 °C to evaluate the thermal stability and decomposition. While we have done DSC in the temperature range of −80 °C to 310 °C mainly to understand the phase transitions and heat capacity but NOT to evaluate the decomposition as we have already evaluated decomposition study using TGA. We have mentioned that the AAILs showed phase transition between 5–170 °C having a peak at around 96–108 °C. The ranges and values were reported by considering all the four AAILs.

Marzena *et al.* commented that “Literature inspection pointed out that the discussed values of specific isobaric heat capacity of [EMIm][BF_4_] and [BMIm][NTf_2_] are related to 298.15 K. Thus, the comparison of the presented specific isobaric heat capacity of AAILs to those of [EMIm][BF_4_], [BMIm][NTf_2_], as well as of water and ethylene glycol is not justified.”

In our work, the “normalized heat capacity (J g^−1^ °C) *versus* temperature” data was analyzed using the “TA Instruments TRIOS software” from the DSC analysis of “normalized heat flow (W g^−1^) *versus* temperature” data. The specific heat capacity at high temperatures is crucial for heat transfer fluids. Because, it is necessary for a HTF to have high specific heat capacity at high temperatures in order to maximize the thermal energy storage capacity and also to reduce the temperature fluctuations while absorbing or releasing heat. We agree that we did not mention the temperature while mentioning the specific heat capacity of [EMIm][BF_4_] and [BMIm][NTf_2_]. However, the mentioned values are not very far from the actual values in a wide temperature range.

For instance, it is reported in the literature by Hasen *et al.* that the specific heat capacity of [EMIm][BF_4_] is in the range of 1.1 to 1.8 J g^−1^ K^−1^ in the temperature range of 293 K to 360 K.^14^

Similarly, it is reported in the literature by Hamidova *et al.* that the specific heat capacity of [BMIM][NTF_2_] is in the range of 1.315 J g^−1^ K^−1^ to 2.0 J g^−1^ K^−1^ in the temperature range of 273.15 K to 413.15 K.^1,15^ Gomez *et al.* have reported the specific heat capacity of [bmim][NTF_2_] as 1.25 J g^−1^ K^−1^ to 1.34 J g^−1^ K^−1^ in the temperature range of 298–333 K.^16^ Similarly, as per literature reports, the isobaric specific heat capacity of water is approximately between 4.2 – 4.8 J g^−1^ K^−1^ in a temperature range of 298–373 K. The highest specific heat capacity at ∼100 °C exhibited by [emim][Arg] (∼14 J g^−1^ °C) and [bmim][Arg] (∼12 J g^−1^ °C) could be due to the large size of anion (Arginate) and complex network of cation–anion interactions due to the presence of strong intermolecular forces such as hydrogen bonds.

Marzena *et al.* have compared the viscosity and density of [EMIm][Gly] reported by us with some other literature values in Table 3.

In our original article, we did not perform a separate analysis for water content in the AAILs as the FTIR did not show any –OH peaks. The comparative Table 3 of Marzena *et al.* clearly shows the measurement techniques used for viscosity are different by different authors.

We have used a stress controlled Modular Compact Rheometer (MCR302, Anton Paar, USA) to perform the rheology studies. Typically, Anton Paar Rheometers (MCR series) are considered as better choice for R & D and advanced testing especially for complex fluids due to their more reliable and accurate measurements.

The other authors, as reported by Marzena *et al.* in Table 3 have used techniques such as Stabinger viscosimetry and Brookfield viscosimetry. The Stabinger viscometer and Brookfield viscometer are ideal for fast and more suitable for routine kinematic viscosity measurements in quality control applications.

Furthermore, the viscosity values as tabulated by Marzena *et al.* in Table 3 clearly shows the discrepancies in the viscosity measurements by the Stabinger viscometer and Brookfield viscometer. For instance, the viscosity of the fluid will get reduced with increase in concentration of water. However, the data reported in Table did not seem to follow this. For instance, the viscosity of IL with respect to water concentration should follow the order of ηIL (<100 ppm) > ηIL (296 ppm) > ηIL (1625 ppm) > ηIL (14400 ppm). However, the reported order of viscosity is ηIL (1625 ppm) > ηIL (14400 ppm) > ηIL (<100 ppm) > ηIL (296 ppm).

Marzena *et al.* commented about the discrepancy between the viscosity variation at different shear rates for [BMIm][BF4]-based INFs reported by us (Chandra *et al.*) with Cherecheş *et al.* in the Fig. 6 of their comment article.^1,17^

We feel that one point which is causing the discrepancy or confusion in Fig. 6(a) and (b) of Marzena *et al.* is the scale of *X*-axis (shear rate) and *Y*-axis (viscosity) of the two plots in the original articles of ours and Cherecheş *et al.*'s.^1,17^ Fig. 6(a) which is ours, the *Y*-axis viscosity scale is from 60 to 200 with increment of 20 for major ticks. While that for Fig. 6(b) of Cherecheş *et al.* has a scale of zero to 1200 with increment of 200 for major ticks.^17^ If we re-scale our plot to match the scale of Cherecheş *et al.*, the viscosity *versus* shear rate of [bmim][BF_4_] will appear as perfectly horizontal line as shown in Fig. 1 below. The shear rate *versus* viscosity of pure [bmim][BF_4_] now matches with the Cherecheş *et al.*^17^

Further, the viscosity of [bmim][BF_4_] as reported by creches *et al.* was ∼100 mPa s. Our values as reported in Fig. 3(a) and 6(a) of our paper are 100 mPa S at 298 K (in viscosity *versus* temperature plot) and 87 mPa s (in viscosity *versus* shear rate plot). We feel that this is a close match considering the differences in the type of instruments used as it is not necessary that every research group has access to same instrument model.

Further, Creches *et al.* have used logarithmic scale for *X*-axis (shear rate) (Fig. 6 of Creches *et al.*) and we have used linear scale for *X*-axis (Fig. 6(a) of Chandra *et al.*, our article).^1,17^ Our Fig. 6(a) by changing the *X*-axis scale to logarithmic scale would look like as shown in Fig. 2 below.

As we have used linear scale and we observed almost constant (or a very minimum change) in viscosity from a shear rate of 100 s^−1^ (as per re-drawn Fig. 1 presented above and Fig. 6 of our original article), we have mentioned the INFs following Newtonian behavior. However, we agree that when *X*-axis changed to logarithmic scale, the Newtonian nature of pure [bmim][BF_4_] and non-Newtonian nature of INFs are more pronounced.

However, we would like to highlight that Marzena *et al.* have wrongly re-plotted the Fig. 6 of Creches *et al.*^17^ The *X*-axis of Fig. 6 of Creches *et al.* has logarithmic scale.^17^ But, Marzena *et al.* have plotted the “same data points on a linear scale” in their Fig. 6(b). This discrepancy can be clearly observed when these two graphs (Fig. 6 of Creches *et al.* and Fig. 6(b) and 4 of Marzena *et al.*) are compared.

Marzena *et al.* claimed while referring to Creches *et al.* that the MWCNT incorporated INFs should exhibit non-Newtonian behavior while the INFs reported by us indicate Newtonian behavior.

We have mentioned that the INF behavior as Newtonian behavior because from a shear rate of 100 s^−1^, no significant deviation was observed in viscosity (Fig. 6(a) of our article). However, as mentioned above, when the *X*-axis is set to logarithmic scale, the non-Newtonian nature is more pronounced.

Furthermore, it is reported in the literature that the non-Newtonian behavior of INFs is usually more pronounced at higher concentrations of MWCNT (>0.1 wt%). The Fig. 6(a) of our article is actually in correspondence with the literature as it can be clearly seen that the non-Newtonian behavior kept increasing with increase in MWCNT concentration. However, we have not stated 0.1wt% MWCNT INFs as non-Newtonian, because the change in viscosity was not significant from a shear rate of 100 s^−1^.

Furthermore, the very significant viscosity jumps at shear rate of “1 s^−1^”, as reported in the Fig. 6 of Creches *et al.* for low concentrations of MWCNT (0.025 wt% to 0.1 wt%) appears surprising. For instance, viscosity of pure IL at shear rate of 1 s^−1^ was 100 mPa.S while that for 0.1 wt% MWCNT incorporated INF was 1200 mPa.S at the same shear rate of 1 s^−1^. It is also strange in Fig. 6 of Creches *et al.* that from the shear rate of ∼600 s^−1^ to 1000 s^−1^, the viscosity of INFs for all MWCNT concentrations were very close (almost coinciding) to that of pure IL. But it is surprising to see that Marzena *et al.* have not highlighted this point.

In this context, the viscosity values of [bmim][BF_4_]+MWCNT INFs reported by us appear more practical than Cherecheş *et al.* Because our results clearly show a gradual increase in viscosity of INFs with increase in weight percent of MWCNT as compared to the viscosity of pure IL at all shear rates in the range of 1 s^−1^ to 1000 s^−1^ (Fig. 6(a) of Chandra *et al.*).

## Conclusions

In conclusion we hope we have provided the necessary clarifications and justifications for the methods used and the results produced. We strongly feel that the properties of AAILs and INFs discussed in our original are accurate and reliable. The only correction is the newly observed non-Newtonian nature of the INFs when the scales of shear rate and viscosity are changed that too at shear rates below 100 s^−1^ above which the behavior of INFs is Newtonian only.

## Conflicts of interest

There are no conflicts to declare.

## Data Availability

No new data were generated or analyzed in this study, and therefore, a data availability statement is not applicable.
